# Evaluation of Perioperative Risk Factors for Infection by Multidrug-Resistant Bacteria in Patients Undergoing Liver Transplantation

**DOI:** 10.3390/jpm15060240

**Published:** 2025-06-10

**Authors:** Rafael Ramos Fernández, Alberto Calvo García, Ainhoa Fernández Yunkera, Silvia Ramos Cerro, Ignacio Garutti, Javier Hortal Iglesias, Patricia Muñoz García, Sergio García Ramos, Adoración Elvira Rodríguez, Mercedes Power Esteban, Patricia Duque González, Patricia Piñeiro

**Affiliations:** 1 Department of Anesthesiology and Resuscitation, Gregorio Marañón University Hospital, 28007 Madrid, Spain; 2 Instituto de Investigación Sanitaria Gregorio Marañón (ISGM), 28007 Madrid, Spain; 3 Department of Digestive Diseases, Gregorio Marañón University Hospital, 28007 Madrid, Spain; 4 School of Medicine, Universidad Complutense de Madrid, 28040 Marid, Spain; 5 Department of Microbiology and Infectious Diseases, Gregorio Marañón University Hospital, 28007 Madrid, Spain

**Keywords:** multidrug-resistant, bacterial infection, perioperative risk factors, liver transplantation

## Abstract

**Background:** Liver transplantation (LT) is a critical intervention for patients with end-stage liver disease. Infections caused by multidrug-resistant bacteria (MDRB) significantly worsen post-transplant outcomes. The main objective of this study was to analyze perioperative risk factors associated with MDRB infections within six months following LT. **Methods:** A retrospective analysis was conducted on 133 medical records of patients who underwent liver transplantation between October 2018 and May 2022. Data collected included the presence of MDRB colonization and infection, as well as various perioperative variables. These were analyzed to identify potential risk factors for MDRB infection and colonization. **Results:** Univariate analysis identified several perioperative variables associated with MDRB infection within six months after LT. Multivariate logistic regression revealed that pre-transplant MDRB colonization (OR 5.72, 95% CI 1.7–18.7, *p* = 0.005) and the requirement for dialysis during postoperative ICU stay (OR 6.42, 95% CI 1.7–23.4, *p* = 0.009) were independent risk factors for developing MDRB infections. MDRB infection occurred in 9.4% of patients and was not significantly associated with increased mortality (*p* = 0.126). **Conclusions:** These findings contribute to a better understanding of the epidemiology and pathophysiology of MDRB infections in the postoperative period of liver transplantation. This knowledge is essential for developing effective prevention and treatment strategies that may improve outcomes in this patient population.

## 1. Introduction

Solid organ transplant (SOT) is the best therapeutic option for patients diagnosed with end-stage organ disease. The median survival of both recipients and grafts has also significantly increased during the last years [1]. Liver transplantation (LT) is an established treatment for patients with acute liver failure and advanced liver disease, with or without hepatocellular carcinoma [1]. Despite advancements in surgical techniques, novel immunosuppressive drugs, vaccination strategies, and improved perioperative care, and by the optimization of prophylactic regimens against opportunistic infections, infections in LT recipients remain the leading cause of morbidity and mortality, with rates ranging between 30% and 60% [2].

Therefore, while the incidence of infections (including opportunistic ones such as cytomegalovirus) is decreasing due to better prevention, the burden of “classical” infections linked to multidrug-resistant (MDR) bacteria especially related to Gram-negative bacilli (GNB) is constantly increasing [2,3].

Bacterial infections are the leading cause of post-transplant infectious complications, accounting for up to 70% of cases, followed by viral and fungal infections [3]. Postoperative bacterial infections frequently lead to graft dysfunction and impair extrahepatic organ function, often resulting in prolonged stays in the intensive care unit (ICU). These complications may necessitate adjustments to immunosuppressive regimens, thereby increasing the risk of graft rejection.

Over the past decade, the incidence of infections caused by multidrug-resistant bacteria (MDRB) in transplant recipients has increased substantially, representing a significant clinical challenge with serious consequences for both graft survival and overall patient outcomes [4].

Repeated and unavoidable exposure to healthcare environments and antibiotics, combined with immunological dysfunction, places liver transplant (LT) recipients at a significantly increased risk of colonization and infection by MDRB. Risk factors for fecal carriage include comorbid conditions, previous hospitalization, urinary tract infections, previous antibiotic therapy, and international travel from high endemic areas [5,6]. In the hospital, risk factors seem to be dependent on local prevalence, recent use of β-lactams or fluoroquinolones, prolonged hospitalization, urinary catheter, transfer from long-term care facilities, and immunosuppression [7]

Identifying specific risk factors and MDRB infection rates at each transplant center is crucial for developing targeted preventive strategies aimed at improving outcomes in LT recipients. One such strategy involves the implementation of Antimicrobial Stewardship Programs (ASPs), designed to optimize antibiotic use and mitigate the emergence of resistance in nosocomial infections. At our center, ASPs have been in place since 2018 [8,9].

The aim of this study is to identify perioperative risk factors associated with MDRB infection and colonization during the first six months following liver transplantation, and to evaluate their impact on patient and graft survival within the framework of an established antimicrobial stewardship program.

## 2. Materials and Methods

### 2.1. Study

This is a retrospective study that includes a systematic review of the medical records of patients who underwent liver transplantation between October 2019 and May 2022. The study was approved by the Ethics Committee of the Gregorio Marañón General University Hospital in Madrid, Spain (approval code: MDR-TXH, approval date: 24 April 2024). The authors declare no conflicts of interest. Clinical data were obtained from hospital records using the HCIS (Hospital Clinical Information System) and Servolab (Microbiology Data Management) platforms.

We excluded patients who died in the operating room or within the first 24 h of admission to the postoperative intensive care unit (PICU). Only one patient was excluded for this reason. During the study period, all implants were orthotopic and whole-organ. No living donor donations occurred, including those involving sequential transplantation. An ex vivo perfusion machine was used on three occasions to maintain and verify liver function prior to implantation. Patient consent was waived due to retrospective design and no interventionism.

### 2.2. Data Records

Preoperative variables included age, sex, weight, height, liver disease etiology and stage, hemoglobin level, platelet count, international normalized ratio (INR), activated partial thromboplastin time (aPTT), fibrinogen, D-dimer, bilirubin, creatinine, high-sensitivity cardiac troponin I (hs-cTnI), and pro-B-type natriuretic peptide (proBNP).

Intraoperative variables included the following: number of red blood cell units transfused, platelet units, fibrinogen, fresh frozen plasma, total volume of crystalloids, donor age, surgery duration (minutes), plasma disappearance rate (PDR) of indocyanine green after arterial reconstruction, lowest hemoglobin during LT, transfusion of more than four units of red blood cells, cold ischemia time, hepatic artery and portal vein flow, and hypotension during the pre-anhepatic (Phase I), anhepatic (Phase II), and post-reperfusion (Phase III) phases defined as mean arterial pressure (MAP) < 60 mmHg for more than 15 min.

Post-reperfusion syndrome was defined as a >30% drop in MAP relative to baseline values, lasting more than 5 min. The need for intraoperative norepinephrine and intraoperative blood transfusion were also recorded.

At the beginning and end of the surgery, the following hemodynamic parameters were collected: heart rate (HR), MAP, central venous oxygen saturation (SvO_2_), temperature, cardiac index (CI), global end-diastolic index (GEDI), pulmonary vascular permeability index (PVPI), and extravascular lung water index (ELWI).

Postoperative variables collected during the PICU stay included the following: hemoglobin level, highest creatinine within the first five postoperative days, aspartate aminotransferase, bilirubin, hs-cTnI, proBNP, length of PICU stay, days of mechanical ventilation, duration of orotracheal intubation, days with a central venous catheter, postoperative graft function according to the Toronto Classification, norepinephrine requirement in the PICU, reoperation within the first postoperative week, hepatic artery thrombosis, portal vein thrombosis, bile leakage, reintubation, mortality, bacteremia, pneumonia during PICU stay, respiratory failure, kidney failure, and the postoperative use of immunosuppressive drugs (corticosteroids, tacrolimus, basiliximab, or mycophenolate mofetil).

### 2.3. Colonization Record

Microbiological cultures from patients were reviewed from three months prior to transplantation to six months afterward. A patient was considered colonized prior to LT if rectal or nasal swab cultures tested positive for any of the following: methicillin-resistant *Staphylococcus aureus* (MRSA); extended-spectrum beta-lactamase (ESBL)-producing Enterobacteriaceae; carbapenemase-producing Enterobacteriaceae (e.g., *Klebsiella pneumoniae* carbapenemase [KPC], metallo-beta-lactamase [MBL], or OXA-48); vancomycin-resistant *Enterococcus faecium* (VRE); or multidrug-resistant non-fermenting Gram-negative bacilli (*Pseudomonas aeruginosa*, *Acinetobacter baumannii*, *Burkholderia cepacia*, *Stenotrophomonas maltophilia*).

Colonization was also recorded if these organisms were detected in diagnostic cultures (e.g., blood, respiratory samples such as tracheal aspirates or bronchoalveolar lavage, urine, abscesses, or other sterile fluids) obtained at admission to the PICU after transplantation.

### 2.4. Infection by Multi-Resistant Microorganism

Medical records and microbiological results were reviewed for the six-month period following liver transplantation. Patients were considered to have an MDRB infection if they had both a clinical diagnosis of infection and a corresponding positive culture for MRSA, ESBL-producing Enterobacteriaceae, carbapenemase-producing Enterobacteriaceae, vancomycin-resistant *E. faecium*, or multidrug-resistant non-fermenting Gram-negative bacilli (*P. aeruginosa*, *A. baumannii*, *B. cepacia*, *S. maltophilia*), excluding samples collected during the first three days of PICU admission.

### 2.5. Statistical Analysis

For the statistical analysis, a comparison of the variables was made between patients who presented colonization or infection by multi-resistant microorganisms before the liver transplant and after the transplant with those who did not present this colonization/infection either before or after the transplant.

Continuous variables were compared using the non-parametric Mann–Whitney U test, while categorical variables were analyzed using the chi-square test or Fisher’s exact test. Variables with a *p*-value < 0.05 were considered statistically significant.

A logistic regression model was then performed to detect the independent risk factors for the appearance of infection by multi-resistant bacilli after the transplant. All those variables in which a *p* < 0.15 had been observed in the univariate analysis for each dependent variable were introduced into the model. Statistical analysis was performed using SPSS version 21.

## 3. Results

### 3.1. Incidence

The medical records of 133 patients who received liver transplants between October 2018 and May 2022 were reviewed. Thirteen patients (9.8%) developed an infection caused by MDRBs within the first six months following liver transplantation. The mean age of the patients was 55 years. We found no association between demographic variables, stage of disease, and preoperative variables and the risk of infection (Table 1).

Fifty-nine patients had hospitalizations lasting more than two days in the three months prior to transplant (42.7%); two more patients (1.5%) had multiple admissions, but none lasted longer than two days. Sixty-five patients received antibiotic treatment for more than three days in the three months prior to transplant (47.1%). Any patient was in ICU three months before LT.

Thirty-four patients (25.6%) received a liver from a controlled asystole donation according to the Maastricht-3 protocol. Ninety-nine patients (74.4%) received an organ from a brain death donation.

### 3.2. Colonization

Twenty-one patients (15%) presented colonization by at least one MDRB before transplant. Of these, 17 (12.3%) presented positive isolates from rectal sample, 4 (2.9%) presented positive isolates from nasal sample, and 1 (0.7%) had positive isolates from rectal and nasal samples. After liver transplantation, 27 patients (20%) presented positive isolates from rectal sample or in cultures of other microbiological samples. The isolated bacteria are shown in Table 2.

No patient colonized by multi-resistant *P. aeruginosa* was detected before and after transplant. Only one nasal sample was found positive for MRSA in a patient who had already been previously colonized. The increase in postoperative colonization by MDRB was due to an increase in the frequency of appearance of bacteria producing carbapenemases (from 10 isolates in 8 patients (5.8% of patients) to 20 isolates in 15 patients (10.9% of patients)) and vancomycin-resistant *E. faecium* which increased from 2 to 7 isolates.

### 3.3. Multi-Resistant Infection After Transplantation

We only found one infection by MDRB during the admission of patients in PICU for immediate postoperative treatment of liver transplantation. It was a pneumonia caused by multidrug-resistant *A. baumanii*. The infectious complications recorded in the first six months after transplantation were reviewed. In the hepatology ward another 12 infectious complications caused by MDRB were recorded. A total of 13 patients (9.4%) presented infectious complications due to MDRB.

### 3.4. Risk Factors for Multidrug-Resistant Infection

A univariate analysis of perioperative, intraoperative (Table 3), and PICU-stay variables identified 19 factors potentially associated with MDRB infection within the first six months following liver transplantation. Patients infected by MDRB had received more fibrinogen, red blood cells, or platelets transfusion intraoperatively than patients with no MDRB infection. Patients with MDRB infection also exhibited higher levels of ProBNP prior to liver transplantation and elevated hs-cTnI levels either at the conclusion of the procedure or during the first day of their PICU stay.

Postoperatively, we observed that AST, INR, and hs-cTnI levels on the first postoperative day were higher in patients who developed postoperative MDRB infections. Additionally, these patients experienced longer PICU and hospital stays, a higher incidence of sepsis, respiratory failure, or dialysis requirements, and poorer postoperative graft function compared to patients without MDRB infections (Table 4).

Then, a multivariate analysis of the indicated parameters was performed. We found that pre-transplant colonization with MDRB (OR 5.72 CI 1.7–18.7 *p* = 0.005) and the use of dialysis during PICU stay (OR 6.42 CI 95% 1.7–23.4; *p* = 0.009) were an independent risk factor to develop MDRB infection in the postoperative period up to six months after transplant.

### 3.5. Survival

**Figure 1:**
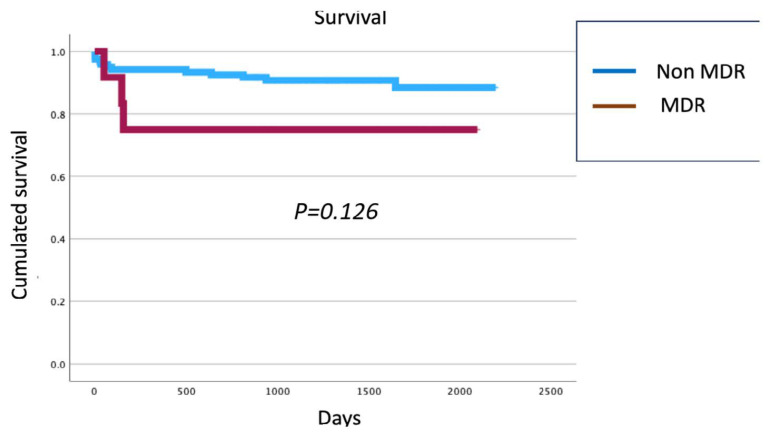
Cumulated survival patients with multidrug-resistant microorganisms infection vs. no infection.

Finally, patients who developed MDRB infections showed no differences in survival compared to those without MDRB infections (Log-rank = 0.126) (Figure 1).

## 4. Discussion

In this study, we identified several independent risk factors associated with the occurrence of multidrug-resistant bacteria (MDRB) infections during the first six months following liver transplantation (LT). Our findings highlight the relevance of pre-transplant colonization and the requirement for renal replacement therapy in the immediate postoperative period. Other contributing factors were also observed.

Pre-transplant colonization is a well-established independent risk factor for MDRB infection within six months of LT [10]. This underscores the critical importance of routine screening for MDRB colonization. The prevalence of MDRB colonization remains one of the most impactful unresolved healthcare challenges [11]. Gram-negative bacteria have been identified as the predominant pathogens [12]. The incidence of colonization among LT candidates is high and exerts a significant influence on postoperative outcomes [13]. Consequently, strategies aimed at preoperative decolonization and/or preventing progression from colonization to infection are essential, particularly in immunocompromised patients such as liver transplant recipients.

There is currently uncertainty about whether MDRB colonization is an indirect marker of patient frailty or severity, indicating a need for a higher number of antibiotics, with the consequent intestinal dysbiosis, or whether colonization physiopathologically affects the clinical course of patients.

Kidney injury following LT is a relatively common complication, with its incidence varying depending on the classification criteria used. Studies have shown that it is associated with 30-day mortality, graft dysfunction, and overall mortality [14,15]. The development of acute kidney injury in the postoperative period is multifactorial; however, patients with renal failure, particularly those requiring dialysis, are at a significantly higher risk of infection by MDRBs [16]. This increased risk necessitates greater use of antibiotics and prolonged hospital stays, thereby enhancing exposure to MDRBs [17]. Notably, a high prevalence of infections caused by MDRBs, such as vancomycin-resistant enterococci and methicillin-resistant *Staphylococcus aureus*, has been reported in dialysis patients [18].

In our study, we did not observe significant differences in baseline renal function prior to LT between patients who developed MDRB infections within six months post-LT and those who did not. However, pre-LT MELD scores were higher, albeit not significantly, in patients who later developed MDRB infections. The MELD score is widely used to assess the severity of chronic liver disease and serves as a predictive tool for mortality among patients on the LT waiting list. This score incorporates INR, bilirubin, and creatinine levels. While preoperative creatinine is a recognized risk factor in many types of surgical interventions, this association has not been consistently demonstrated in transplant surgery patients [15,19]. Moreover, our cohort showed a higher incidence of sepsis, which may have substantially contributed to the development of acute kidney injury. Both the direct effects of sepsis on renal function and the necessity of broad-spectrum antibiotics to treat severe infections in immunosuppressed patients could explain why a greater proportion of MDRB-infected patients in our study required renal replacement therapy during their stay in the PICU.

We also observed significant intraoperative and immediate postoperative hemodynamic differences in patients who developed MDRB infections. These individuals exhibited more pronounced myocardial dysfunction, as reflected by cardiac output, elevated hs-cTnI, and proBNP levels.

To date, no prior studies have established a direct relationship between MDRB infections and myocardial function. However, existing evidence suggests a strong association between myocardial stress and the development of non-cardiac postoperative complications. Noordzij and colleagues reported a higher incidence of sepsis in non-cardiac surgeries among patients with elevated hs-cTn levels. It has been hypothesized that the perioperative stress response which includes inflammatory activation, sympathetic and neuroendocrine hyperactivity, immunosuppression, and hypercoagulability [20], in conjunction with perioperative complications such as bleeding-anemia, hypovolemia-tachycardia, and hypoxia, may initiate or exacerbate myocardial cell injury [21,22]. Furthermore, myocardial injury, characterized by diastolic dysfunction and reduced cardiac output, may impair the ability to meet the increased perfusion demands of extracardiac tissues affected by surgical trauma. Based on these findings, we propose that a causal relationship is plausible whereby patients with MDRB colonization are predisposed to developing infections by these microorganisms, which carry an elevated risk of severe complications.

Surgical manipulation of the biliary tract during LT increases the risk of bile contamination, which may contribute to MDRB infection. Sugawara et al. found that 7.5% of patients undergoing hepatectomy with bile duct resection had MDRB-related biliary colonization or infection, with identical strains isolated from surgical wounds and blood cultures [23]. Zhong et al. similarly reported higher rates of biliary complications in patients with MDRB infections (30.3% vs. 4.6%) [24]. In our study, MDRB-infected patients had nearly fourfold higher rates of bile leakage, which likely contributed to infection.

Reintervention for bile leaks emerged as an independent risk factor for MDRB infection within six months post-LT, consistent with previous reports linking bile leakage to increased bacterial infections. This association may be due to prolonged abdominal drainage, which increases the risk of surgical site infections [25]. In addition, bile leakage is known to extend hospital stays, thereby increasing MDRB exposure. Portal vein thrombosis (PVT) in non-tumoral patients also necessitates longer operative times and greater transfusion requirements, and has been associated with worse 1- and 5-year survival [26]. These factors may further predispose patients to postoperative MDRB infection, as do other complications such as bile leakage.

In our study, we did not find that the presence of pneumonia was a risk factor for infection by MDRB infections, as has been reported in other studies. However, patients who developed MDRB infections in the postoperative period of LT exhibited a higher incidence of documented respiratory failure. Although the duration of mechanical ventilation was longer in patients with MDRB infections, this difference did not reach statistical significance. We believe that with a larger sample size, statistical significance might have been achieved. We hypothesize that these patients underwent more extensive airway manipulation to improve their respiratory status, which could explain the colonization of the airway by MDRBs and the subsequent development of infections caused by these microorganisms during the late postoperative period. Additionally, airway manipulation and the increased risk of pneumonia in these immunocompromised patients may have led to the prophylactic use of antibiotics, potentially contributing to the deleterious development of MDRB.

Intraoperative blood transfusion was identified as an independent risk factor for MDRB infection in our study, aligning with previous reports. This may be due to increased iron availability, which facilitates bacterial growth and virulence by overcoming host defenses [27]. In fact, in the face of acute infections or injuries, the body responds by sequestering iron as a defense mechanism, and with transfusion, the effect of this defense is counteracted. In addition, allogeneic blood transfusion leads to the exposure of alloantigens in the circulation, which gives rise to an immunomodulatory effect with decreased action of NK cells, decreased CD4/CD8 ratio, and alterations in antigen presentation or in cellular immunity [28]. However, although blood transfusion appears to play an immunosuppressive role, it is important to highlight that it constitutes an indirect marker of greater intraoperative complications and more complex and prolonged procedures that are associated with a higher risk of infectious complications.

Contrary to other studies [29,30], MDRB infections in our cohort were not associated with reduced survival. However, it is essential to recognize that MDRB-infected patients often present with additional mortality risk factors, including severe liver and kidney dysfunction, advanced age, graft dysfunction, biliary complications, and perioperative bleeding.

The impact of multidrug-resistant infections on liver transplant patients highlights the importance of knowing the ecology of each center, even each unit, in order to establish the most appropriate prevention and early treatment measures.

A better understanding of the factors influencing the development of infectious complications caused by multidrug-resistant microorganisms after liver transplant may help identify patient groups that could benefit from personalized preventive or therapeutic interventions.

We recognized that our study has several limitations, including a small sample size and its retrospective design. The data were collected from the hospital database, and any gaps in data collection during patient care may have influenced the results. Therefore, prospective studies with larger populations are needed to draw definitive conclusions regarding risk factors. Additionally, the experience of a single center may differ significantly from that of other countries or regions globally. It is also important to note that part of the study period coincided with the COVID-19 pandemic, which led to enhanced hospital hygiene measures and a reduced number of liver transplants. As a result, further research is needed to investigate future trends in MDRB infections in patients undergoing liver transplantation.

In conclusion, this study enhances our understanding of the epidemiology and pathophysiology of MDRB infections in the postoperative period following liver transplantation. This knowledge may inform effective prevention and treatment strategies to improve outcomes in this vulnerable patient population.

## 5. Conclusions

- Infections caused by multidrug-resistant bacteria (MDRB) following liver transplantation are clinically significant and impact postoperative outcomes.
- Both preoperative and intraoperative risk factors contribute to the development of these infections.
- Pre-transplant colonization with MDRB is a well-established and independent risk factor.
- Perioperative cardiac and renal function are modifiable factors that influence infection risk.
- Identifying these modifiable risk factors may help define specific patient phenotypes who could benefit from personalized preventive or therapeutic strategies.

## Figures and Tables

**Table 1:** Preoperative variables: demographic, preoperative laboratory parameters and scores for the stage of disease.

	NO-MDRB (n = 120) Median (IQ25–IQ75)	MDRB (n = 13) Median (IQ25–IQ75)	*p* Value
Age (y)	58 (50–63)	61 (56–63)	0.410
Sex (male) (n, %)	87 (70.2)	7 (53.8)	0.228
Weight (kg)	75 (65–87)	70 (60–72)	0.253
Height (cm)	168 (164–174)	162 (159–174)	0.217
Hemoglobin (g/dL)	12.2 (10.4–13.7)	11.2 (10.2–13.3)	0.347
Platelets per microliter ×10^3^	79 (55–109)	69 (66–134)	0.783
INR	1.28 (1.16–1.56)	1.35 (1.29–1.62)	0.082
aPTT (s)	34.1 (31.6–38.5)	37.9 (33.2–40.4)	0.444
Hs-cTnI before surgery (ng/mL)	3.4 (1.9–5.9)	3.1 (1.7–3.9)	0.790
D-dimer (µg/mL)	195 (129–793)	500 (203–797)	0.430
Preoperative proBNP (pg/mL)	133 (73–318)	141 (50–276)	0.024
Bilirubin (mg/dL)	2.1 (1–4.3)	2.5 (1.4–11.7)	0.297
Creatinine (mg/dL)	0.8 (0.71–0.98)	0.9 (0.84–1.09)	0.334
Child (points)	8 (6–10)	9 (8–11)	0.076
MELD (points)	13.2 (9.6–17)	16.3 (11.1–19.3)	0.069
Portal Thrombosis (n, %)	26 (32.9)	2 (22.2)	0.407
ETIOLOGY			0.742
Alcohol	40 (33.3)	4 (30.8)	
Virus	38 (31.6)	3 (23.1)	
Cholangitis	11 (9.2)	2 (15.4)	
FHF	7 (5.8)	1 (7.7)	
Cancer	24 (20)	3 (23.1)	

MDRB: Multidrug-resistant Bacteria; INR: International normalized ratio; FHF: Fulminant hepatic failure; aPTT: activated partial thromboplastin time; MELD: Model for end-stage liver disease.

**Table 2:** Bacterias isolated in epidemiological control cultures prior to transplantation or at the time of admission to the Postoperative Intensive Care Unit (PICU), and bacterias isolated in epidemiological control during ICU admission after transplantation.

MDRB Colonization Before Transplantation		MDRB Colonization After Transplantation	
Bacteria	Isolates n = 32	Bacteria	Isolates n = 45
*E. coli* ESBLP	31.25% (10 isolates)	*E. coli* ESBLP	22.2% (10 isolates)
*K. pneumoniae* ESBLP	9.3% (3 isolates)	*K. pneumoniae* ESBLP	8.8% (4 isolates)
*K. pneumoniae* CBP	15.6% (5 isolates)	*K. pneumoniae* CBP	8.8 % (4 isolates)
*E. coli* CBP	6.25% (2 isolates)	*E. coli* CBP	13.3% (6 isolates)
*C. freundii* CBP	6.25% (2 isolates)	*En. cloacae CBP*	15.5% (7 isolates)
*MRSA*	15.6% (5 isolates)	*C. freundii* ESBLP	4.4% (2 isolates)
*VRE*	6.26% (2 isolates)	*VRE*	15.5% (7 isolates)
Others (*P. mirabilis* ESBLP. *E. cloacae* CBP, and *S. maltophilia*)	9.3% (3 isolates)	Others (*C. amalonaticus* ESBLP. *K. oxytoca* CBP. *A. baumanii*, MRSA and *S. maltophilia*)	11.1% (5 isolates)

*E. coli*: *Escherichia coli*; ESBLP: extended-spectrum beta-lactamase-producing enterobacteriaceae; *K. pneumoniae*: *Klebsiella pneumoniae*; CBP: carbapenemase-producing organism; *C. freundii*: *Citrobacter freundii*; MRSA: Methicillin-resistant *Staphylococcus aureus*; VRE: vancomycin-resistant *Enterococcus faecium*; *P. mirabilis*: *Proteus mirabilis*; *S: Stenotrophomonas*; *C. amalonaticus*: *Citrobacter amalonaticus*; A: *Acinetobacter*.

**Table 3:** Intraoperative variables recorded. Quantitative values are presented as median (25% and 75% quartiles) and Qualitative values are presented as total number (% of patients).

	Total	Non-Infected Patients (n = 120)	Infected Patients (n = 13)	*p* Value
Blood transfusion during LT	64 (46.7)	55 (44.4)	9 (69.2)	0.078
Concentrated red blood cells units	0 (0–3)	0 (0–2)	3 (1–5)	0.018
Transfusion of more than 4 units of blood	16 (11.7)%	12 (9.7)	4 (30.8)	0.036
Platelets units	0 (0–0)	0 (0–0)	0 (0–1)	0.049
Fibrinogen (gr)	0 (0–4)	0 (0–3)	4 (0–4)	0.048
Fresh frozen plasma units	0 (0–0)	0 (0–0)	0 (0–0)	0.322
Total crystalloids (mL)	2325 (2000–2500)	2300 (2000–2500)	3000 (2400–3000)	0.322
Hs-cTnI before surgery (ng/mL)	3.4 (1.8–5.8)	3.4 (1.9–5.9)	3.1 (1.7–3.9)	0.790
Hs-cTnI 60 min after reperfusion (ng/mL)	19.6 (12.4–35.1)	18.8 (12.4–34.7)	31.3 (23.5–49.7)	0.033
Preoperative proBNP (pg/mL)	132(73–283)	133 (73–318)	141 (50–276)	0.024
Donor age (y)	62 (51–73)	62 (51–73)	69 (60–74)	0.238
Graft Ischemia length (min)	357 (312–397)	357 (315–396)	358 (308–420)	0.919
Hepatic Flow artery (mL/min)	200 (140–300)	200 (145–300)	165 (128–265)	0.311
Portal Flow venous (mL/min)	1115 (765–1600)	1200 (850–1600)	950 (685–1627)	0.443
Surgery duration (min)	200 (178–232)	200 (180–231)	233 (170–260)	0.699
PDR 60 min after reperfusion (%)	15.2 (11.2–19)	15 (11–19.2)	17.1 (12.4–18.1)	0.649
Lower hemoglobin during LT (gr/dL)	7.6 (6.8–9.8)	7.6 (6.8–9.8)	7.3 (6.5–8.4)	0.722
Hypotension Phase I	37 (27.2)	33 (26.8)	4 (30.8)	0.493
Hypotension Phase II	33 (24.3)	29 (23.6)	4 (30.8)	0.39
Hypotension Phase III	53 (39)	49 (39.8)	4 (30.8)	0.374
PRS	33 (24.3)	27 (22)	6 (46.2)	0.053
Use Norepinephrine during surgery	59 (43.4)	51 (41.5)	8 (61.5)	0.165
HR_baseline (bpm)	68 (59–79)	68 (59–78)	66 (63–82)	0.773
MAP_baseline (mmHg)	75 (66–87)	74 (66–87)	80 (74–91)	0.743
CI baseline (L/min/m^2^)	3.1 (2.67–3.66)	3.1 (2.7–3.7)	3.17 (2.6–3.35)	0.363
GEDI_baseline (mL/m^2^)	639 (543–725)	639 (539–725)	610 (581–670)	0.823
HR_end of surgery (bpm)	82 (70–94)	80 (70–95)	82 (71–86)	0.773
MAP_end of surgery (mmHg)	63 (55–70)	62 (51–68)	61 (55–70)	0.594
GEDI_end of surgery (mL/m^2^)	609 (508–691)	677 (585–830)	799 (668–821)	0.517
CI_end of surgery (L/min/m^2^)	4.27 (3.2–5.1)	3.68 (3.1–4.4)	3.14 (2.3–4.7)	0.037

LT: Liver transplantation; Hs-cTnI: High sensitivity cardiac troponin I; ProBNP: Pro-B-type natriuretic peptide; PDR: Plasma disappearance rate; PRS: Post-reperfusion syndrome; HR: Heart rate; MAP: Mean arterial pressure; CI: Cardiac index; GEDI: Global end diastolic index.

**Table 4:** Postoperative variables recorded in the study. Values are presented as median (25% and 75% quartiles). We highlight those values that we consider relevant after the analysis.

	Total (n = 133)	Non-MDRB Infection (n = 120)	MDRB Infection (n = 13)	*p* Value
Hb (g/dL) PICU arrival	10.1 (8.8–11.75)	10.05 (8.8–11.7)	10.3 (9–11.95)	0.589
Highest creatinine in the first 5 days PO	1.43 (1.01–1.94)	1.39 (1.01–1.9)	2.03 (1.1–2.46)	0.152
AST day 1 after LT	144 (85–262)	133 (81–230)	326 (163–631)	**0.002**
AST day 2 after LT	69.2 (36.2–169)	66 (35–152)	166 (56–546)	**0.018**
INR day 1 after LT	1.93 (1.58–2.51)	1.91 (1.56–2.42)	2.74 (1.83–4.57)	**0.009**
INR day 2 after LT	1.51 (1.28–1.89)	1.5 (1.28–1.87)	1.98 (1.24–3.01)	0.215
Bilirubin day 1 after LT	3.6 (2.2–6.8)	3.2 (1.9–8.1)	3.65 (2.15–6.75)	0.806
Bilirubin day 2 after LT	1.9 (1–3.2)	1.9 (1.1–3.4)	2.15 (0.8–4.75)	0.898
Troponin I day 1 after LT	66.2 (30.4–156)	59 (27.8–123)	138 (86.4–225)	**0.024**
Troponin I day 2 after LT (ng/mL)	87.9 (32.9–253)	77 (31–249)	161 (118–265)	0.238
ProBNP on 1st day PICU	226 (102–384)	219 (98–365)	334 (208–589)	0.225
PICU stay (days)	2 (1–3)	2 (1–3)	3 (2–7)	0.075
Reintubation (Yes)	11 (8)	9 (7.3)	2 (15.4)	0.305
Pneumonia in PICU (Yes)	1 (0.7)	0 (0)	1 (7.7)	0.95
Respiratory failure (Yes)	43 (32.3)	35 (29.1)	8 (61.5)	**0.034**
Length Mechanical Ventilation (days)	0 (0–1)	0 (0–1)	1 (0–2)	0.077
Length intubation (days)	0 (0–1)	0 (0–1)	1 (0–2)	0.081
Reintervention (Yes)	37 (27.8)	32 (26.7)	5 (38.5)	0.274
Retransplantation (Yes)	15 (11.3)	12 (10)	3 (23.1)	0.165
Length intravenous central line (days)	2 (1–3)	2 (1–3)	3(2–7)	0.072
Hospital stay length (days)	16 (13–23)	15 (13–14)	28 (16–48)	**0.004**
PICU stay length (days)	3 (2–4)	3 (2–4)	4 (3–7)	**0.021**
Postoperative Graft Function 72 h (I vs. II, III, or IV grades)	36 (29)	36 (26.3)	0 (0)	**0.021**
Extubated patient in the operating room	89 (66.9)	84 (70)	5 (38.5)	0.057
Norepinephrine need in PICU	19 (14.3)	17 (14,2)	2 (15.4)	0.598
Reintervention in first week	22 (23.7)	19 (22.6)	3 (23.1)	0.36
Died in first week post LT	2 (7.1)	2 (7.7)	0 (0)	0.86
Death in PICU	4 (3.2)	4 (2.9)	0 (0)	0.651
Died in first year post LT	10 (7.5)	7 (5.8)	3 (23.1)	0.059
Bacteremia in PICU	1 (0.7)	1 (0.8)	0 (0)	0.137
Other infection in PICU	26 (19.7)	21 (17.5)	5 (41.7)	0.059
Sepsis	7 (5.3)	3 (2.5)	4 (30.7)	**<0.001**
PICU respiratory failure	31 (23.5)	25 (20.8)	6 (46.1)	**0.035**
Hemodialysis	17 (12.9)	12 (10)	5 (38.4)	**0.009**
Readmission PICU	5 (3.8)	3 (2.5)	2 (16.7)	0.065
Arterial thrombosis	8 (6.1)	6 (5)	2 (16.7)	0.156
Portal thrombosis	6 (4.5)	4 (3.3)	2 (16.7)	**0.035**
Bile leakage	15 (11.4)	11 (9.2)	4 (33.3)	**0.031**
Cholangitis	5 (3.8)	3 (2.5)	2 (16.7)	0.065
Corticosteroids	130 (96.3)	117 (95.2)	13 (100)	0.598
Tacrolimus	69 (87.3)	63 (87.5)	6 (46.2)	0.892
Basiliximab	98 (71.5)	87 (70.2)	11 (84.6)	0.272
Mycophenolate mofetil	101 (73.7)	91 (73.4)	10 (76.9)	0.54
MDRB colonization before LT	22 (16.5)	17 (14.2)	5 (38.5)	**0.028**
MDRB colonization at PICU	28 (21)	21 (17.5)	7 (53.8)	**0.005**

Hb: Hemoglobin; PICU: Postoperative Intensive Care Unit; LT: Liver transplantation; MDRB: Multidrug-resistant bacteria.

## Data Availability

The original contributions presented in this study are included in the article. Further inquiries can be directed to the corresponding author.

## Abbreviations

MDRB: Multidrug-resistant microorganisms; LT: Liver transplantation; ICU: Intensive care units; hs-cTnI: High sensitivity cardiac troponin I; PDR: Plasma disappearance rate; MAP: Mean arterial pressure; proBNP: Pro-B-type natriuretic peptide; HR: Heart rate; CI: Cardiac index; GEDI: Global end diastolic index; PICU: Postoperative intensive care unit; MRSA: Methicillin-resistant S. aureus; ESBLPE: Extended-spectrum beta-lactamase-producing enterobacteriaceae; CPE: Carbapenem-producing enterobacteriaceae; KPC: Klebsiella pneumoniae carbapenemase; MBC: metallo-beta-lactamase; OXA-48: oxacillinase 48; VRE: vancomycin-resistant *Enterococcus faecium*. *P*.: *Pseudomonas*; *A*.: *Acinetobacter*; *St*.: *Stenotrophomonas*; INR: International normalized ratio; FHF: Fulminant hepatic failure; aPTT: Activated partial thromboplastin time; MELD: Model for end-stage liver disease; *E. coli*: *Escherichia coli*; Pr: Proteus; PRS: Post-reperfusion syndrome; NK: Natural killer.
